# Ag/Cu-Chitosan Composite Improves Laundry Hygiene and Reduces Silver Emission in Washing Machines

**DOI:** 10.3390/polym15030695

**Published:** 2023-01-30

**Authors:** Mengdan Qiang, Jianrong Wu, Hongtao Zhang, Xiaobei Zhan

**Affiliations:** Key Laboratory of Carbohydrate Chemistry and Biotechnology, The Ministry of Education, Jiangnan University, Wuxi 214122, China

**Keywords:** chitosan, laundry, silver emission, antibacterial material

## Abstract

Textiles can be contaminated with pathogens during household laundering, potentially leading to human sickness. In this work, chitosan (CTS) was used as a substrate to prepare Ag/Cu-CTS composite, which was applied in laundering and showed a remarkable antibacterial effect on *Escherichia coli* and *Staphylococcus aureus*. The mechanical strength of Ag/Cu-CTS composite beads was higher than 400 MPa. The Ag/Cu-CTS composite were further characterized by scanning electron microscopy and energy dispersive spectroscopy. This composite had a strong inhibitory effect on several laundry pathogens, such as *Acinetobacter* sp., *Pseudomonas aeruginosa*, and *Candida albicans*. Using a standard laundering program and 15 g of Ag/Cu-CTS composite beads, the antibacterial rates reached 99.9%, and no silver emission was detected, thereby satisfying the Chinese requirement for washing machines. After 160 runs of laundering tests, this composite still has an excellent antibacterial effect. For the first time, chitosan is successfully applied as an antibacterial material on household electric appliances.

## 1. Introduction

Harmful microorganisms are ubiquitous in the human living environment. One of our frequently used electric appliances, the washing machine, has favorable conditions for the growth of microorganisms and has been proven to be ideal habitat for harmful pathogens. These microorganisms originate from laundry textiles or breed in washing machines, resulting in cross-contamination between laundry and washing machines [1]. Several reports have analyzed the microbial communities of domestic washing machines and tested the potential effects of these microorganisms on laundry [2,3,4]. Nix et al. found that the common prokaryote in the washing machines was *Proteobacteria*, and the common eukaryotes were *Basidiomycota* and *Ascomycota* [5]. Stapleton et al. investigated the formation of odor inside the washing machine and its influence on the odor of clothes [6]. The results indicated that microbes could transfer from the drum and rubber seals of the washing machine to the laundry, leading to malodor on the washing machine and laundry. Callewaert et al. reported that bacteria associated with water, skin, and clothing, as well as bacteria associated with biofilm synthesis, could undergo microbial exchange during laundering in domestic washing machines [7]. Among them, *Escherichia coli* and *Staphylococcus aureus* are common strains in washing machines, and they are also the test strains required by China’s national standards as antibacterial indicators of washing machines. Laundry and washing machine microbiota may even affect the user’s skin microbiota.

The laundering efficiency of washing machines depends on the mechanical circulation of the washing drum, the flushing of the fabrics with water and detergent, and the appropriate washing time and temperature [8]. Energy efficiency and water saving in the laundering process are important for low-carbon living and environmental protection [9]. Sustainable laundering methods include washing at lower temperatures, reducing water, and using bleach-free detergents. However, these methods cannot effectively remove harmful microorganisms inside the washing machine and on the laundry, thereby negatively affecting laundry hygiene. The existing germ-killing and sterilizing technologies of washing machines have shortcomings, such as high energy consumption and pollutant discharge. Therefore, the development of germ-killing materials has received a lot of attention.

**Scheme 1:**
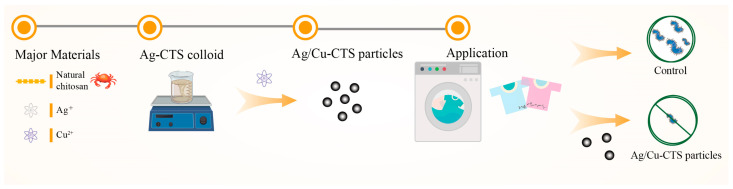
Preparation and application of Ag/Cu-CTS composite.

Chitosan is the main product after the deacetylation of chitin, and it is a natural and excellent functional polymer material with excellent biocompatibility and biodegradability [10]. Importantly, chitosan has good antibacterial activity and heavy metal adsorption capacity [11]. However, the low solubility of chitosan limits its antibacterial activity. The common method to improve the germ-killing capacity of chitosan is to modify chitosan or combine it with other antibacterial materials [12,13]. The special structure of chitosan enables it to chelate with metal ions under certain conditions [14], and adding metals to chitosan can greatly improve its antibacterial activity [15,16]. For instance, Khan et al. prepared a chitosan–zinc complex by irradiation; it could not only effectively inhibit a variety of bacteria, but also show excellent antifungal activity [17]. Masayuki et al. prepared a chitosan/silver material with an effective inhibitory effect on microorganisms by adsorbing nano-silver on chitosan [18]. In addition, a Cu–chitosan nanoparticle complex could stick to the cell wall, penetrate the cell wall, and cause bacterial death, and the antibacterial activity of this complex against *Escherichia coli* was higher than that of the chitosan [19]. The general design and concept are shown in Scheme 1. The Ag/Cu-CTS composite showed excellent antibacterial effect and negligible Ag emission in laundering. To our knowledge, it is the first time that chitosan has been used as an antimicrobial material in washing machines for textile disinfection.

## 2. Materials and Methods

### 2.1. Chemicals and Strains

Chitosan (deacetylation degree 80–95%), AgNO_3_, cupric acetate, glutaraldehyde (25% aqueous solution), acetic acid, NaOH, dibutyl phthalate, glycerol, sodium citrate, sodium borohydride (NaBH_3_), and ethanol absolute were purchased from Sinopharm Chemical Reagent Company (Shanghai, China). *Escherichia coli* was obtained from American Type Culture Collection (ATCC 25922). *Staphylococcus aureus* (ATCC 6538) was obtained from Shanghai Bioresource Collection Center (Shanghai, China). *Candida albicans*, *Pseudomonas aeruginosa*, and *Acinetobacter* sp. were isolated from a contaminated washing machine and stored at Jiangnan University.

### 2.2. Preparation of Ag/Cu-CTS Composite

First, 40 mL of chitosan solution (4%, dissolved in 2% acetic acid) was mixed with 1 mL of AgNO_3_ of different concentrations (0.05–0.25 g/mL) for 30 min at room temperature. After that, three different reducing agents (glycerol, sodium citrate, and sodium borohydride) were added separately to the reaction system. A sample without a reducing agent was used as a control. The molar ratio of the reducing agent to silver nitrate was 1.5:1. Then, the mixture was incubated at 60 °C for 30 min. As a result, Ag-CTS colloids were obtained. The Ag-CTS colloid was characterized by an ultraviolet-visible spectrophotometer (UV-Vis, U-3900, Hitachi, Japan), transmission electron microscope (TEM, H-7650, Hitachi, Japan), and X-ray diffractometer (Bruker Daltonics, Billerica, MA, USA).

Subsequently, 7.2 g of chitosan and 0.4 g of cupric acetate were dissolved in 60 mL acetic acid (4%, *v*/*v*) by sonication. Then, the chitosan solution was mixed with the prepared Ag-CTS colloid (40 mL). Subsequently, 2.5 mL of dibutyl phthalate was added, and the mixture was incubated for 5 min. Then, 10 mL of glutaraldehyde solution was added, and the mixture was agitated for 10 min at different temperatures. When the solution became a gel-like lump, an appropriate size gel bead was made into granules (around 4–5 mm in diameter), which were immersed in 20 g/L NaOH solution for 3 h. After washing with ultrapure water, the granules were soaked in ethanol solution (50%, *v*/*v*) for 2 h. Finally, after washing and drying, the Ag/Cu-CTS composite was obtained.

### 2.3. Characterization of Ag/Cu-CTS Composite Particles

#### 2.3.1. Mechanical Strength

The prepared Ag/Cu-CTS composite particles were dried at 60 °C to a constant weight. Then, the mechanical strength of these antibacterial particles was measured using a particle strength testing machine (KQ-2, Nanjing Kehuan Analytical Instrument Co., Ltd., Nanjing, China). The particles were measured five times to obtain the average value.

#### 2.3.2. Antibacterial Properties

*E. coli* and *S. aureus* were cultivated in 3 mL of LB medium in a 14 mL tube set at 37 °C and 220 rpm. Then, the culture was diluted 1000 times, and 0.2 mL bacterial suspension was spread evenly on the LB agar plates which contained one antibacterial particle in the center of the plate. The plate was cultivated at 37 °C for 12 h, and the size of the inhibition zone was recorded. In addition, three representative pathogenic bacteria, *Candida albicans*, *Pseudomonas aeruginosa*, and *Acinetobacter* sp., were used to test the antibacterial effect using LB agar plates.

#### 2.3.3. Scanning Electron Microscope (SEM) and Energy Dispersive Spectroscopy (EDS)

The morphology of the prepared Ag/Cu-CTS composite was observed. The sample was placed on conductive plastic. After gold spray treatment, the sample was placed into the SEM (JMS-IT800, Hitachi, Japan) to observe the surface morphology of the sample. EDS (JMS-IT800, Hitachi, Japan) was performed to confirm the presence of copper and silver in the samples.

### 2.4. Application of Ag/Cu-CTS Composite Particles in Laundry

#### 2.4.1. Loss of Antibacterial Properties

A sample of 15 g Ag/Cu-CTS composite was packed in a plastic mesh bag and fixed at a lower position in a washing machine (TB80V80WDCLG, Wuxi Little Swan Electric Co. Ltd., Wuxi, China). After 10 runs of standard laundering, the Ag/Cu-CTS composite particle was subject to antibacterial properties determination. The loss of antibacterial properties of this composite was evaluated by the size of the inhibition zone.

#### 2.4.2. Antibacterial Effect Detection

The antibacterial effect detection of Ag/Cu-CTS composite particles was measured according to China National Standard GB 21551.5-2010. In summary, 1 mL of bacterial suspension of *S. aureus* and *E. coli* (1.0 × 10^9^–9.0 × 10^9^ CFU/mL) was poured onto the cotton textile (100 mm × 100 mm, three pieces). After drying, the cotton textiles were laundered in a washing machine (TB80V80WDCLG) using a standard program. Subsequently, the test sample textiles were taken out, and the bacteria count method was used to measure the number of viable bacteria remaining on the test sample textile. The cotton textile with bacterial suspension was set as the control group. The antibacterial rate can be calculated as 100 × (N1 − N2)/N1 (N1: number of residual bacteria in the control group after washing; N2: number of residual bacteria in the test group after washing).

#### 2.4.3. Detection of Silver Emission

Samples of 5, 10, and 15 g of Ag/Cu-CTS composite particles were placed in a washing machine. Discharge samples were obtained from the water outlet when a standard laundering program was completed. Silver emission in outlet wastewater was measured by an atomic absorption spectrophotometer (AA-240, Varian, Palo Alto, CA, USA).

### 2.5. Statistical Analysis

Three independent replicates were performed for all experiments. Statistical data analysis was performed with T-texts with SPSS 25.0. *p* values of <0.05 were considered statistically significant, and statistical significance is indicated as * for *p* < 0.05 and ** for *p* < 0.01.

## 3. Results

### 3.1. Preparation and Structural Characterization of Ag-CTS Colloid

**Figure 1:**
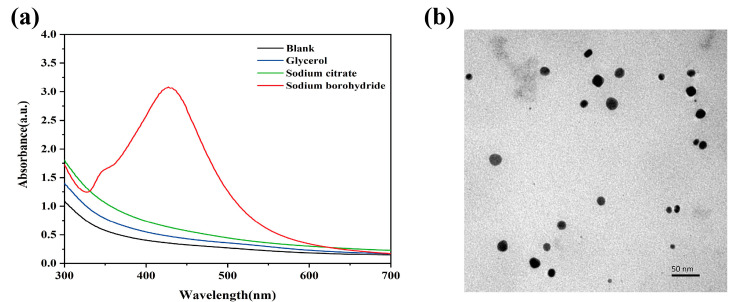
Characterization of Ag-CTS colloid. (**a**) UV-Vis of Ag-CTS colloid. (**b**) TEM of Ag-CTS colloid (the reducing agent is sodium borohydride).

Firstly, the liquid-phase reduction method was adopted to reduce AgNO_3_ to prepare Ag-CTS composite colloid. In order to verify the strength of the reducing capacity of different reducing agents on the silver nanoparticles, three common reducing agents, glycerol, sodium citrate, and sodium borohydride, were selected for the test. The effects of different reducing agents on Ag-CTS colloids are shown in Figure 1a. Ag^+^ in the reaction mixture is able to coordinate with the abundant -NH_2_ in chitosan. With the addition of a reducing agent, Ag^+^ in the Ag-CTS colloid was reduced to Ag^0^. In the group with the sodium borohydride, the Ag-CTS colloid had an obvious absorption peak around 430 nm, which was the characteristic absorption peak of Ag [20]. The other groups did not have obvious absorption peaks, which were similar to the control. The reason might be that only a small amount of Ag^+^ was reduced due to the low efficiency of the reducing agent, and the Ag content may not reach the detection limit after dilution. Meanwhile, the Ag-CTS composite colloid prepared with sodium borohydride as a reducing agent has the smallest polymer dispersity index (Table S1) among the composite colloid prepared with different reducing agents. Therefore, sodium borohydride was chosen as a reducing agent for the preparation of Ag-CTS composite colloid. Further observation by TEM (Figure 1b) showed that the prepared Ag-CTS colloid was nearly spherical, with a relatively uniform particle size of 20 nm [21].

**Figure 2:**
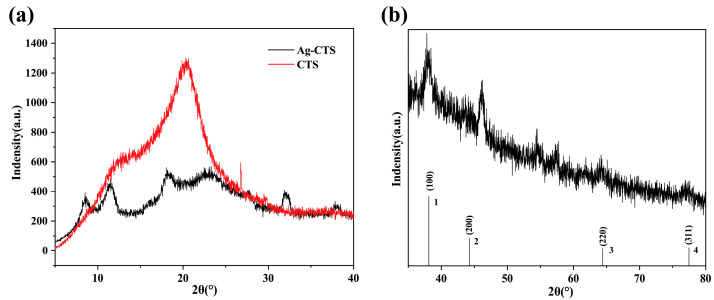
XRD analysis of CTS and Ag-CTS colloid. (**a**) XRD analysis of CTS and Ag-CTS colloid. The red curve is CTS, and the black curve is Ag-CTS colloid. (**b**) XRD analysis of Ag-CTS colloid.

Then, we further characterized the prepared chitosan colloid. The X-ray diffraction patterns of chitosan and Ag-CTS colloid is shown in Figure 2. Compared with chitosan, the peak position of the Ag-CTS colloid changed remarkably, and the diffraction peak intensity corresponding to the II crystal form decreased. The possible reason is that chitosan and Ag^+^ undergo chelation, which reduces the intermolecular hydrogen bond, and thus decreases the crystallinity. In Figure 2b, Ag in the Ag-CTS colloid had four obvious absorption peaks at 2θ of 36.00, 46.20, 64.40, and 77.32. The peak positions of Ag were consistent with the data on JCPDS card 04-0783. Peak 1, peak 2, peak 3, and peak 4 correspond to the (111), (200), (220), and (311) crystal planes of cubic silver, respectively, indicating that this substance is elemental silver of a cubic system [22]. The diffraction peak of this curve is relatively sharp, indicating that the colloids have good crystallinity.

### 3.2. Preparation of Ag/Cu-CTS Composite

**Figure 3:**
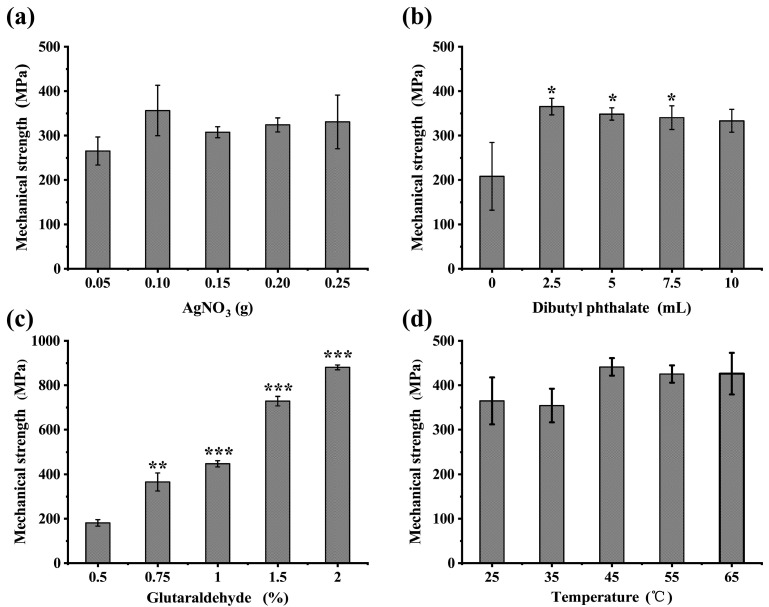
Effect of preparation conditions on the mechanical strength of Ag/Cu-CTS composite. (**a**) Effect of AgNO_3_ additions on the mechanical strength of Ag/Cu-CTS composite. (**b**) Effect of dibutyl phthalate additions on the mechanical strength of Ag/Cu-CTS composite. (**c**) Effect of glutaraldehyde additions on the mechanical strength of Ag/Cu-CTS composite. (**d**) Effect of temperature on the mechanical strength of Ag/Cu-CTS composite. Data are shown as the mean ± SD of triplicates with good reproducibility. The SD is represented as error bars. Statistically significant differences are indicated: * for *p* ≤ 0.05, ** for *p* ≤ 0.01, and *** for *p* ≤ 0.001.

In the washing machine, the antibacterial material has to withstand the impact of turbulence in the laundering process, and mechanical strength is a crucial requirement. Therefore, the prepared Ag-CTS colloid is not suitable for use in washing machines. To solve this problem, we prepared the Ag-CTS colloid in pellet form, and Cu ions were added to further increase the antibacterial activity. The effect of AgNO_3_ mass, plasticizer, cross-linking agent, and temperature on the mechanical strength of the Ag/Cu-CTS composite was evaluated, as shown in Figure 3. When the added AgNO_3_ was 0.05 g, the mechanical strength of the composite particles was the lowest (264.9 MPa). With increased Ag, the mechanical strength of the composite particles increased. The mechanical strength reached the maximum (356.1 MPa) when 0.10 g of AgNO_3_ was added. Though a higher silver content could improve the mechanical strength of the composite material, an economic dosage is preferred. The amount of plasticizer (dibutyl phthalate) could affect the mechanical strength of the Ag/Cu-CTS composite, as shown in Figure 3b. The mechanical strength was the lowest without any plasticizer (208.1 MPa). With the addition of a plasticizer, the mechanical strength of the Ag/Cu-CTS composite increased. The reason might be that dibutyl phthalate acts as a plasticizer, which has adhesiveness and water resistance. Glutaraldehyde was used as a cross-linking agent to prepare Ag/Cu-CTS composite. As shown in Figure 3c, the mechanical strength of the composite increased significantly with the increase in glutaraldehyde concentration (*p* < 0.05). In the case of 0.5% glutaraldehyde, the mechanical strength of the composite was at least 181.7 MPa. The mechanical strength of the composite reached as high as 881.2 MPa with the addition of 2% glutaraldehyde. The amino and hydroxyl groups in chitosan are easily reacted with glutaraldehyde, resulting in improved mechanical strength of the chitosan-based Ag/Cu-CTS composite [23]. Finally, the cross-linking temperature could also affect the mechanical strength of the Ag/Cu-CTS composite, as shown in Figure 3d. When the temperature increased from 35 °C to 45 °C, the mechanical strength of the composite increased from 354.2 MPa to 441.2 MPa. Higher temperatures may affect the cross-linking efficiency, resulting in higher mechanical strength of the Ag/Cu-CTS composite.

### 3.3. Plate Inhibition Zone of Ag/Cu-CTS Composite

**Figure 4:**
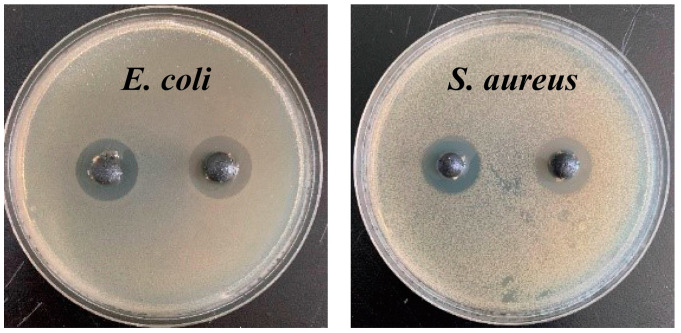
Inhibition zone of Ag/Cu-CTS composite against *E. coli* and *S. aureus.*

Subsequently, to test the antibacterial properties of the prepared pellets, the typical *E. coli* and *S. aureus* were chosen for testing. The experimental results are shown in Figure 4 and Table 1. All the tests achieved high efficiency in the antibacterial properties of the composite. With the increase in silver content, the size of the inhibition zone increased significantly. In the case with 0.05 g AgNO_3_, the diameter of the inhibition zone of the composite against *E. coli* and *S. aureus* was 8.1 and 5.4 mm, respectively. When the AgNO_3_ mass was increased to 0.20 g, the diameter of the inhibition zone of *E. coli* and *S. aureus* reached 16.0 and 9.1 mm, respectively. In the following study on characterization, the Ag/Cu-CTS composite was prepared using 0.20 g AgNO_3_. However, other preparation conditions had no significant effect on the size of the inhibition zone, which may be due to the similar Ag^+^ diffusion on the agar plates.

### 3.4. Structural Characterization of Ag/Cu-CTS Composite

**Figure 5:**
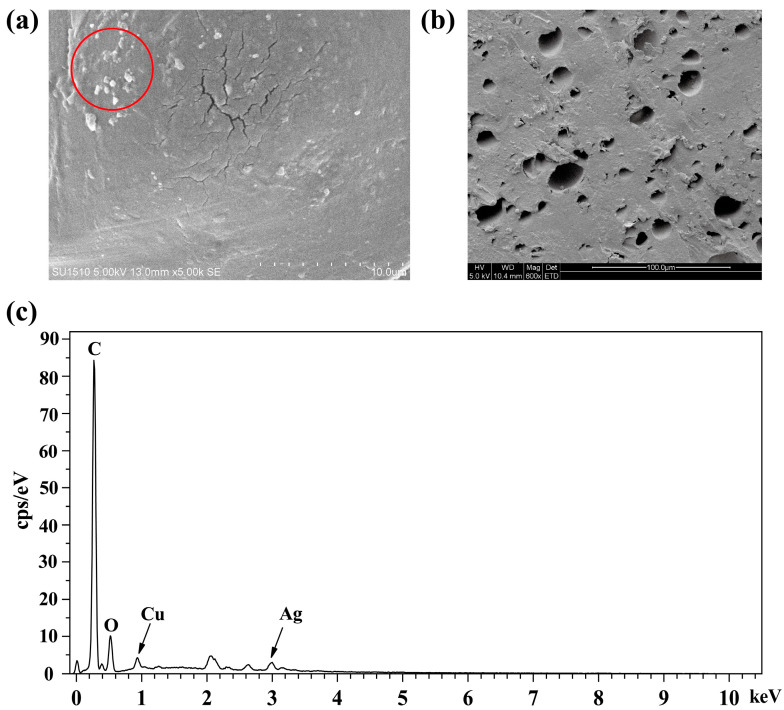
SEM and EDS of Ag/Cu-CTS composite. (**a**) SEM of surface of Ag/Cu-CTS composite. (**b**) SEM of cross section of Ag/Cu-CTS composite. (**c**) EDS of the surface of Ag/Cu-CTS composite.

The surface and cross-section of Ag/Cu-CTS composites were further observed by SEM, as shown in Figure 5a. This composite was relatively flat, with a uniform and dense structure, and many particles exist on the surface. These white particles on the surface could be Ag and Cu elements [24]. The cross-section of the composite (Figure 5b) showed a rough crater structure. Such rough surface morphology may be due to insufficient cross-linking of chitosan. The crater structure of the composite could provide more adsorption sites for the binding to Ag^+^ and Cu^2+^ [25]. Figure 5c shows the energy-dispersive spectroscopy (EDS) spectra of the Ag/Cu-CTS composite. In the left part of the spectrum, several peaks located between 0.2 keV and 3 keV can be clearly observed. The maxima located on the left part of the spectrum at 0.2 keV and 0.5 keV confirm the presence of carbon and oxygen, respectively. The hardly visible maxima located near 1 keV and 3 keV indicate the presence of copper (4.45%) and silver (12.08%), respectively. The carbon (67.97%) and oxygen (15.50%) peaks in the samples examined confirm the presence of the chitosan matrix.

### 3.5. Inhibition of Laundry Pathogens by Ag/Cu-CTS Composite

**Figure 6:**
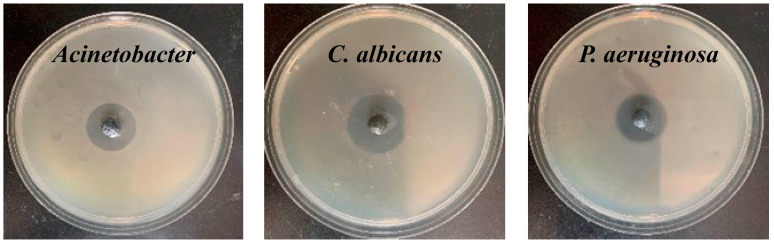
Inhibition zone of Ag/Cu-CTS composite against common pathogenic bacteria from washing machines.

A large number of studies on microbial contamination in laundering have shown that the representative bacteria species in washing machines at the phylum level are *Proteobacteria.* Jacksch et al. examined the composition of bacterial communities in washing machines and found that *Proteobacteria* accounted for 85.8% at the phylum level; it was the major phylum in washing machines [8]. Chris et al. also detected a large number of *Proteobacteria* on washed cotton fabrics [7]. *Acinetobacter* sp. and *Pseudomonas aeruginosa* belong to *Proteobacteria*, which were common pathogenic bacteria in washing machines. *Candida albicans* is also a typical pathogen fungus that exists in the washing machine. In this work, the antibacterial activity against *C. albicans*, *P. aeruginosa*, and *Acinetobacter* sp. was evaluated using Ag/Cu-CTS composite, as shown in Figure 6. The inhibition zone diameters of Ag/Cu-CTS composite against *Acinetobacter*, *C. albicans*, and *P. aeruginosa* were 13.1, 16.0, and 12.2 mm, respectively. These data showed that this composite had a favorable inhibitory effect on a variety of pathogenic bacteria and can exhibit a favorable antibacterial effect in the complicated laundering environment.

### 3.6. Antibacterial Activity Loss Test of Ag/Cu-CTS Composite

Generally, germ-killing materials have to function in washing machines for a long time, and the working life of antibacterial materials is critical for the application. With the increasing runs of laundering, the diameter of the inhibition zone of the composite decreased, but the reduction was not apparent, as shown in Table 2. When the run of laundering was 20, the size of the inhibition zone was 12.2 mm. The size of the inhibition zone is 8.7 mm at the laundering runs of 80. Until the laundering run of 160, the size of the inhibition zone was still 7.3 mm. Although the diameter of the inhibition zone decreased after multiple runs of laundering, the Ag/Cu-CTS composite still had good antibacterial properties. This finding indicates that this composite has a long-term effect during application.

### 3.7. Silver Emission in Laundering

The amount of antibacterial material also has an important impact on the antibacterial effect in laundering. Using more Ag/Cu-CTS composite could increase the antibacterial rate, as shown in Table 3. The antibacterial rate reached 99.9% by adding 15 g of Ag/Cu-CTS composite in the laundering process. Then, the silver content in the drainage of the washing machine was further measured. Due to the low concentration of silver ions, no silver ions were detected in the discharge water. Furthermore, the ability of chitosan to adsorb silver ions may also contribute to it. As a result, the application of Ag/Cu-CTS composite in laundering did not generate silver emission pollution.

## 4. Discussion

Laundry hygiene is problematic in the laundering process using household washing machines for consumers. Thus far, several germ-killing methods are developed, such as ozone treatment, high-temperature sterilization, and silver ion elimination. Among these, Ag-silica glass particles are used widely, but Ag^+^ emission pollution is inevitable. Therefore, chitosan, a natural polysaccharide with a well-known antibacterial capacity, has become an excellent option. In this work, the Ag/Cu-CTS composite showed an excellent antibacterial effect in the laundering procedure. Several chitosan-based blend films and porous scaffolds showed lower mechanical strength (<100 MPa) and were difficult to use in laundering [26,27]. Hua et al. found that the antibacterial rate of the CTS/Carbon-Ag composite particle was above 80% after soaking the composite in water for 50 d [28]. The mechanical strength of the Ag/Cu-CTS composite was higher than 400 MPa and could withstand water flushing in washing machines. After 160 runs of the laundering test, this composite showed the same physical dimension.

Silver ion is an excellent and widely-used inorganic antibacterial agent, which can bind the sulfhydryl group on bacterial protease, inactivate bacterial protease, and cause cell death. Li et al. found that the diameter of the inhibition zone of CTS-Au/Ag against *E. coli* and *S. aureus* was about 1.25 times that of CTS/Ag, whereas CTS and CTS-Au showed no evident inhibition zone. The cell integrity of bacteria treated with CTS-Au/Ag was destroyed, showing shrinkage and mucus morphology [29]. Pinto et al. used citrate and borohydride reduction methods to prepare colloidal silver nanoparticles and prepared a CTS composite material, which has excellent antibacterial activity against *S. aureus*, *K. pneumoniae*, and *E. coli* [30]. In our work, the antibacterial activity of the Ag/Cu-CTS composite is better than that of the sole Ag-CTS composite. The inhibition zone sizes of Ag-CTS composites against *E. coli* and *S. aureus* in Figure S1 were 7.6 mm and 7.1 mm, respectively, which were lower than those of Ag/Cu-CTS composites with the same silver content. These results indicate the synergistic antibacterial action of Ag^+^ and Cu^2+^.

The structure of chitosan is rich in active amino groups and hydroxyl groups, which enable its chelating capacity on metal elements. In recent years, many CTS-based materials are developed to adsorb heavy metals and other toxic compounds in wastewater. Razzaz et al. prepared chitosan/TiO_2_ composite nanofibers and chitosan/TiO_2_ composite adsorbents and found that the composite fibers could remove Cu^2+^ and Pb^2+^ in the wastewater effectively [31]. Among them, the CTS/TiO_2_ composite adsorbent had a higher metal adsorption capacity than that of TiO_2_-coated CTS nanofibers. Singh et al. designed a chitosan-based sulfide polymer to remove arsenic from polluted water, and this polymer could remove 85.4% of As^3+^ and 87% of As^5+^ in the groundwater [32]. In this work, the Ag/Cu-CTS composite can inhibit bacteria in the laundry effectively. Meanwhile, excess Ag^+^ may be adsorbed to the chitosan particles and this dual-function strategy can reduce the emission of Ag^+^ in the laundering drainage.

## 5. Conclusions

The Ag/Cu-CTS composite was prepared and successfully used as an antibacterial material in laundering. Under the conditions of AgNO_3_ of 0.20 g, dibutyl phthalate of 2.5 mL, glutaraldehyde of 1.0% (*v*/*v*), and cross-linking at 45 °C, the Ag/Cu-CTS composite showed remarkable antibacterial effect and mechanical strength. Using a standard laundering program and 15 g of Ag/Cu-CTS composite, the antibacterial rates reached 99.9%, and no silver emission was detected. Using a standard laundering program and 15 g of Ag/Cu-CTS composite, the antibacterial rates reached 99.9%, and no silver emission was detected. Therefore, it does not cause silver ion contamination. After long-term laundering tests, this composite still has an excellent antibacterial effect. Ag/Cu-CTS composite also has a strong inhibitory effect on several laundry pathogens, such as *Acinetobacter* sp., *Pseudomonas aeruginosa*, and *Candida albicans*. In the future, Ag/Cu-CTS composite can potentially substitute for Ag-silica glass in washing machines as an antibacterial material.

## Figures and Tables

**Table 1:** Inhibition zone of Ag/Cu-CTS composite under different preparation conditions.

	Preparation Conditions	Diameters of Inhibition Zone against *E. coli* (mm)	Diameters of Inhibition Zone against *S. aureus* (mm)
AgNO_3_ (g)	0.05	8.1	5.4
	0.10	10.3	7.2
	0.15	14.5	8.0
	0.20	16.0	9.1
	0.25	16.0	9.0
Glutaraldehyde (%)	0.5	16.1	8.0
	0.75	16.0	8.3
	1.0	16.2	8.3
	1.5	15.9	8.0
	2.0	16.0	8.1
Dibutyl phthalate (mL)	0	13.8	8.1
	2.5	14.3	8.3
	5.0	12.4	8.2
	7.5	14.0	8.4
	10.0	12.1	7.7
Temperature (°C)	25	14.1	7.3
	35	14.5	8.0
	45	16.2	8.5
	55	16.1	8.0
	65	16.0	8.1

**Table 2:** Residual antibacterial activity of Ag/Cu-CTS composite after long-term laundering.

Runs of Laundering	Diameter of Inhibition Zone against *E. coli* (mm)
0	12.7
20	12.2
40	10.5
60	10.1
80	8.7
100	8.1
120	8.0
140	8.0
160	7.3

**Table 3:** Antibacterial rate of Ag/Cu-CTS composite and silver emission in laundering drainage.

Amount of Ag/Cu-CTS Composite Used for Laundering (g)	Colony (CFU/mL)	Inhibition Rate (%)	Ag Emission
0	2.65 × 10^5^	/	Undetected
5	2.085 × 10^5^	21.3	Undetected
10	5.85 × 10^4^	77.9	Undetected
15	0	99.9	Undetected

## Data Availability

All data are available in the Manuscript.

## Supplementary Materials

The following supporting information can be downloaded at: https://www.mdpi.com/article/10.3390/polym15030695/s1, Figure S1: Inhibition zone of Ag-CTS composite against *E. coli* and *S. aureus*; Table S1: Polymer dispersity index (PDI) of Ag-CTS composite colloid with different reducing agents.
