# Supplementary material for: Ag/Cu-Chitosan Composite Improves Laundry Hygiene and Reduces Silver Emission in Washing Machines
Source: Polymers (Basel). 2023 Jan 30;15(3):695. doi: 10.3390/polym15030695 (PMC9919517; doi:10.3390/polym15030695)
Supplement: Supplementary file 1 [file polymers-15-00695-s001.zip › polymers-2170464-supplementary.pdf]

Supplementary material

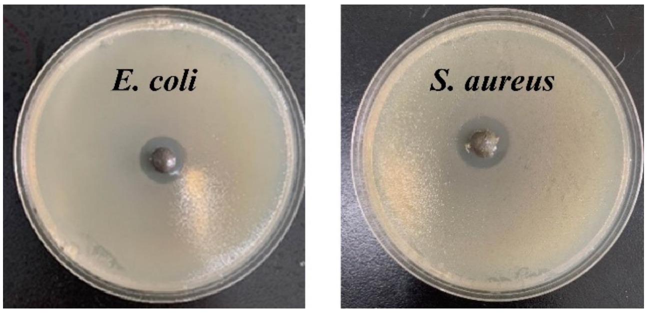

Figure S1. Inhibition zone of Ag-CTS composite against *E. coli* and *S. aureus.*

Table S1. Polymer dispersity index (PDI) of Ag-CTS composite colloid with different reducing agents.

Reducing agents	PDI
Control	0.32
Glycerol	0.27
Sodium citrate	0.42
Sodium borohydride	0.20
